# Level of physical activity of health professionals in a district hospital in KwaZulu-Natal, South Africa

**DOI:** 10.4102/sajp.v71i1.234

**Published:** 2015-08-31

**Authors:** Siyabonga H. Kunene, Nomathemba P. Taukobong

**Affiliations:** 1 Department of Physiotherapy, University of KwaZulu-Natal, Westville Campus, South Africa; 2 Department of Physiotherapy, Sefako Makgatho Health Sciences University (SMU), South Africa

## Abstract

**Background:**

Health professionals have a role to play in the promotion of physical activity in order to prevent the ever-increasing burden of diseases associated with physical inactivity. Determination of the level of physical activity amongst health professionals managing patients presenting with various lifestyle-related conditions is most pertinent.

**Object:**

The purpose of the present study was to ascertain the level of physical activity of health professionals at Estcourt Hospital in KwaZulu-Natal Province.

**Method:**

A cross-sectional survey of 109 health professionals was conducted over a period of three consecutive weeks in 2012. The Global Physical Activity Questionnaire (GPAQ) and other related data such as socio-demographic characteristics was used to collect data. Data were analysed using descriptive and inferential statistics to determine relationships between variables. An analysis guide was used to determine the level of physical activity with reference to the GPAQ guidelines recommended by the Word Health Organization using the metabolic equivalent of task (MET)-minutes per week indicators.

**Results:**

The overall level of physical activity was: 31% of participants were high, with MET-minutes/week ≥ 3000; 29% were moderate, with MET-minutes/week ≥ 600; and 40% were low, with MET-minutes/week < 600. Although black women predominantly reported low levels of physical activity, age was found to be significantly related to the level of physical activity (*p* = 0.000, *r* = −0.637).

**Conclusion:**

An intervention to promote physical activity amongst health professionals is essential to promote healthy living.

## Introduction

Physical inactivity is one of the leading critical risk factors globally for lifestyle-related non-communicable diseases (NCDs) (World Health Organization [WHO] 2004). The WHO (2004) further states that the burden of lifestyle conditions is highest in high-income countries, but a similar profile is emerging in low-income countries, contributing towards rising healthcare costs. Over the past few years, physical activity has been found to be a key health behaviour associated with reduced morbidity and mortality (Lambert & Kolbe-Alexande 2005). Physical activity is important and a cornerstone of health and wellbeing (Andersen *et al.* 2010). Physical activity is defined as any movement of the body that is produced by the skeletal muscles as a result of energy expenditure (Caspersen, Powell & Christenson 1985).

Maintaining personal good health and healthy functioning for daily activities is critical (Lahti *et al.* 2010). Therefore it is important for individuals to ensure that a sufficient intensity of regular physical activity is performed to maintain good health, physical functioning and work activity of a high standard (Lahti *et al.* 2010). A number of health benefits are generally associated with physical activity. These benefits are evident when an individual adheres to exercise principles such as appropriate frequency, duration and intensity of exercise (Lambert & Kolbe-Alexande 2005). Exercise, such as regular aerobic physical activity and strengthening of the musculoskeletal system, has been recommended to improve cardiovascular fitness (Haskell, Bair & Hill 2009). Physical activities, such as simple walking, jogging, swimming, cycling and many more related activities, are good forms of exercise that are effective in improving the individual’s health status (Haskell, Bair & Hill 2009; Nyrop *et al.* 2011). Walking as an ambulatory activity has been confirmed to contribute to the overall physical activity of South African adults (Cook *et al.* 2010). Such activities are reported to improve functional status and prevent limitations concerning physical activity (Nyrop *et al.* 2011). Van Cauwenberg *et al.* (2012), in support of the WHO recommendation, stated that people engaged in moderate-intensity physical activity for 30 minutes at least 5 days per week experience significant benefits, which include a reduction in the age-related risk of chronic disease, especially amongst the growing population of older adults (Van Cauwenberg *et al.* 2012).

A combination of diet, physical exercise and cognitive behavioural training results in significant weight loss, decreased blood pressure and increased aerobic fitness. The resultant benefits are encouraging for individuals, especially in addressing health problems (Christensen *et al.* 2011). Daily physical activity can reduce high blood pressure, lead to improvement in the level of low-density lipoprotein cholesterol and blood glucose in overweight individuals, even without significant weight loss. Physical activity also reduces the chances of developing colon cancer and breast cancer amongst women. It can therefore be concluded that individuals who perform adequate physical activity throughout their lives improve their quality of life (WHO 2004).

Diseases related to physical inactivity are said to contribute to approximately 1.9 million deaths globally each year (WHO 2004). Lack of physical activity can lead to musculoskeletal disorders and pain that can affect the ability to work effectively and even to attend work (Mackey *et al.* 2007). Work-related injuries, especially amongst older workers, have been associated with lack of physical activity, resulting in excessive fatigue and poor productivity (Mackey *et al.* 2007). Globally, low levels of physical activity have been estimated to be responsible for 22% of cases of ischaemic heart disease, 14% of type 2 diabetes mellitus, 16% of colon cancer, 11% of ischaemic stroke and 10% of breast cancer (Joubert *et al.* 2007). A current global comparative risk assessment study (GCRA) reports that 1.92 million deaths and 19 million disability-adjusted life years (DALYs) may be related to physical inactivity. These findings translate into 3.3% of mortality and 1.3% of morbidity worldwide being a result of physical inactivity (Joubert *et al.* 2007).

The South African population, and particularly black people, has moved towards a disease profile very similar to that of high-income countries, where NCDs (mainly chronic and lifestyle-related conditions) are major public health problems (Walter, Du Randt & Venter 2011). South Africa has an estimated 43% – 49% prevalence of physical inactivity amongst adolescents and adults (Micklesfield *et al.* 2014). Chronic lifestyle diseases were responsible for 28.5% of deaths in the Western Cape Province population between the ages of 35 and 64 in 2001 (Lambert, Bohlmann & Kolbe-Alexander 2001). People of younger to older age groups in North-West Province of South Africa were found to be insufficiently active (Lambert & Kolbe-Alexande 2005); only a few were classified as highly active, with most being either inactive or moderately active. The researchers also found that the youth spent more than 3 hours/day watching television and did not participate in physical activities (Lambert & Kolbe-Alexande 2005).

In the developing world, and especially in sub-Saharan Africa, adults including health professionals are not regularly physically active (Nizeyimana & Phillips 2005). Health professionals are expected to be knowledgeable in matters relating to healthy lifestyle and, in their capacity, should be role models for maintaining a healthy lifestyle to the general population. According to Skaal (2011), most health workers in South Africa are overweight and inclining towards obesity. Many South African female health professionals were found to engage in limited health-promoting practices and showed low levels of physical activity and high levels of overweight and obesity (Walter *et al.* 2011). Overweight, obesity, musculoskeletal pain and low physical capacity has been found to result from the unhealthy lifestyle of inactivity amongst most health professionals (Christensen *et al.* 2011; Jonsdottir, Börjesson & Ahlborg 2011; Walter *et al.* 2011).

The purpose of the present study was therefore to determine and describe the level of physical activity amongst health professionals at Estcourt Hospital in KwaZulu-Natal Province.

## Materials and methods

### Design

A cross-sectional survey requesting retrospective information was conducted over a period of three consecutive weeks in November 2012 to measure the level of physical activity amongst Estcourt Hospital health professionals.

### Study population, sampling procedure and sample size

A convenience sampling method was used to select participants for the study. At the time of the study, there were 150 potential participants; however, not all of them couldbe found on the days of data collection, and consequently the eventual sample selected comprised 109 individuals. Those who participated included doctors, nurses, physiotherapists, occupational therapists, dietitians, radiographers, speech therapists, audiologists and paramedics. All ages, races, genders, nationalities and those in full-time employment for at least 6 months at the same hospital in the year 2012 were included. The Raosoft statistical tool recommended a sample size of 109 for a 95% confidence level, and the responding health professionals thus became an acceptable number.

### Instrument

The Global Physical Activity Questionnaire (GPAQ) version 2 of the WHO was modified and used to collect data. Socio-demographics (which included gender, age, race, profession and work experience) were added to the original questionnaire as section A. The GPAQ questions were included as section B. The questionnaire included short questions about participants’ activities at work, movement to and from places, recreational activities and sedentary behaviours. The questionnaire had been recently tested and found valid and reliable by Herrmann *et al.* (2013) for clinical purposes amongst adults. An acceptable evidence of short- and long-term test-retest reliability and modest validity of this tool has been found. Before the data were collected, a pilot study was done amongst 10 health professionals working at Escourt Hospital to ensure validity. No corrections and modifications were indicated by the results of the pilot study.

### Data collection

Ethical clearance was obtained from the MEDUNSA Research and Ethics Committee (MREC), and permission to conduct the study was requested and obtained in writing from Escourt Hospital Chief Executive Officer (CEO). The consenting health professionals were given leaflets containing the purpose and objectives of the study and thereafter requested to sign consent forms. Questionnaires were hand-distributed to participants at their workstations in the hospital. A period of 3 days to complete and return the questionnaire was agreed on with the participants.

### Data analysis

Information from the questionnaire was captured onto a Microsoft Excel spreadsheet, cleaned and imported into the SPSS statistical programme (version 20) for analysis. The level of physical activity was analysed by taking into consideration the metabolic equivalent of task (MET)-minutes per week indicators and the days of physical activity. To calculate MET-minutes per week per participant, the following formula was used: (MET value) × (time of activity in minutes per day) × (days of activity per week) = MET-minutes per week. The overall level of physical activity was categorised as high, moderate or low. Descriptive and inferential (*p* values and Pearson’s correlation) values were calculated as well.

## Results

### Demographic profile

The study yielded a 100% response as all 109 responding health professionals correctly completed and returned the questionnaires. Most of the participants were black (82%) and female (83%), with the nursing profession constituting more than half (57%) of participants, followed by doctors (14%) (Table 1). The remaining professions combined accounted for slightly over a quarter (29%) of the participants. Most (60%) participants were in the ≥ 40-years-old category, and 40% were in the < 40-years-old category. More than half of the participants (54%) had ≤ 5 years of work experience, and just under half (46%) had worked for > 5 years in the same hospital.

**TABLE 1:** Participants’ demographic profiles.

Demographics	Category	*n*	Percentage
Age	< 40	44	40
	≥ 40	65	60
Race	White	9	8
	Indian	8	7
	Mixed race	3	3
	Black	89	82
Work experience	≤ 5 years	59	54
	> 5 years	50	46
Gender	Male	18	17
	Female	91	83
Profession	Doctor	15	14
	Dentist	1	1
	Nurse	62	57
	Physiotherapist	5	4
	Occupational therapist	1	1
	Speech therapist	1	1
	Audiologist	2	2
	Radiographer	4	4
	Dietitian	2	2
	Paramedic	6	5
	Pharmacist	10	9

*N* = 109.

### Level of physical activity

Approximately a third (31%) presented with high levels of physical activity, achieving a mean of 6522 MET-minutes per week (Table 2). Less than a third (29%) presented with moderate physical activity with a mean of 1513 MET-minutes per week, and two-fifths (40%) had low levels of physical activity with a mean of 216 MET-minutes per week. The results also showed that mostly physiotherapists (75%) and paramedics (80%) who participated were within the high level of physical activity, as opposed to other professionals. Mostly doctors (54%), nurses (45%) and radiographers (75%) who participated were in the low level of physical activity (Table 3).

**TABLE 2:** Level of physical activity categories versus metabolic equivalent of task-minutes/week.

Level of physical activity	Percentage	Mean MET-min./week	Normal MET-min./week
High	31	6522	≥ 3000
Moderate	29	1513	≥ 600
Low	40	216	< 600

*N* = 109.

MET, metabolic equivalent of task.

**TABLE 3:** Profession versus level of physical activity.

Profession	Level s of physical activity					
		Low		Moderate		High	
		*n*	%	*n*	%	*n*	%
Audiologist	1	50	1	50	0	0
Dentist	0	0	1	100	0	0
Dietitian	1	50	1	50	0	0
Doctor	8	54	5	33	2	13
Nurse	28	45	17	27.5	17	27.5
Occupational therapist	0	0	0	0	1	100
Paramedic	1	16.6	1	16.6	4	66.6
Pharmacist	1	10	5	50	4	40
Physiotherapist	1	20	0	0	4	80
Radiographer	3	75	1	25	0	0
Speech therapist	0	0	0	0	1	100
Total	44	40	32	29	33	31

*N* = 109.

Table 4 presents the descriptive and correlation test results. Younger participants (< 40 years old) were found to be more active than older participants (≥ 40 years old). A significant correlation between age and level of physical activity (*p* = 0.000, *r* = −0.637) was demonstrated, indicating that as age of participants increased, the level of physical activity decreased. Female participants engaged in all levels of physical activity but the majority (34%) group showed low levels of physical activity. There was no significant relationship between gender and the level of physical activity. Indian participants engaged only in low (4.6%) and moderate (2.8%) levels of physical activity. White participants engaged more in high (4.6%) and less in low (2.8%) and moderate (0.9%) physical activity. The few mixed race participants engaged in low physical activity (0.9%), whilst none fell into the moderate category and more engaged in high levels of physical activity (1.8%). Amongst black participants, an equally low number engaged in low (32.1%), moderate (24.8%) and high (24.8%) levels of physical activity. There was no correlation between race and level of physical activity. Participants with ≤ 5 years’ work experience appeared to be less active than those with > 5 years at Estcourt Hospital. No correlation between work experience and level of physical activity was found.

**TABLE 4:** Correlation between demographics and levels of physical activity.

Demographic	Variables	*n*	Levels of physical activity						Pearson correlation and *p* value
						Low		Moderate		High		
						*n*	%	*n*		%	*n*	%	
Gender	Male	18	6	5	6	5	6	5	r = −0.054
	Female	91	37	34	26	25	28	26	p = 0.579
Age	< 40	44	0	0	20	18.35	24	22.02	r = −0.637
	≥ 40	65	44	40.37	12	11.01	9	8.26	p = 0.000
Race	White	9	3	2.75	1	0.92	5	4.59	
	Black	89	35	32.11	27	24.77	27	24.77	r = −0.116
	Indian	8	5	4.59	3	2.75	0	0	p = 0.229
	Mixed race	3	1	0.92	0	0	2	1.83	
Work experience	≤ 5 years	59	21	19.27	19	17.43	19	17.43	r = −0.089
	> 5 years	50	23	21.1	13	11.93	14	12.84	p = 0.367

*N* = 109.

Table 5 shows specific physical activities in which participants were involved. Nearly two-fifth (38.5%) engaged in vigorous and almost half (49.5%) in moderate work-related physical activities for at least 10 minutes continuously. The results further showed that almost a third (35.8%) of participants walked or cycled between places for at least 10 minutes continuously. Very few engaged in vigorous (23.9%) and moderate (37.6%) sport and moderate, fitness or recreation physical activities. The results in Table 6 show a significant correlation between gender and vigorous work-related (*p* = 0.040, *r* = 0.197) and vigorous sport- and recreation-related (*p* = 0.007, *r* = 0.257) physical activities. Age variable was significantly related to vigorous work-related (*p* = 0.000, *r* = −0.407) and moderate physical activity (*p* = 0.000, *r* = −0.382). The time spent sitting and reclining amongst participants was also found to be significantly related to age (*p* = 0.009, *r* = 0.252).

**TABLE 5:** Physical activities amongst participants.

Physical activities (at least 10 minutes continuously)	Mean responses			
	** **	Yes		No	
	** **	*n*	%	*n*	%
Vigorous work (large increase in breathing/heart rate)	42	38.5	67	61.5
Moderate work (small increase in breathing/heart rate)	54	49.5	55	50.5
Walk or use bicycle	39	35.8	70	64.2
Vigorous sports, fitness or recreation (large increase in breathing/heart rate)	26	23.9	83	76.1
Moderate sports, fitness or recreation (small increase in breathing/heart rate)	41	37.6	68	62.4

*N* = 109.

**TABLE 6:** Correlation between demographics and physical activities.

Demographic	Variables	*n*	Pearson correlation and *p* value					
			** **	** **	** **	Vigorous work	Moderate work	Walking or cycling to and from work	Vigorous sport and recreation	Moderate sport and recreation	Time spent sitting and reclining
Gender	Male	18	r = 0.197	r = −0.095	r = 0.011	r = 0.257	r = 0.093	r = 0.125
	Female	91	p = 0.040	p = 0.327	p = 0.907	p = 0.007	p = 0.034	p = 0.196
Age	< 40	44	r = −0.407	r = −0.382	r = 0.054	r = 0.073	r = 0.097	r = 0.252
	≥ 40	65	p = 0.000	p = 0.000	p = 0.578	p = 0.450	p = 0.314	p = 0.009
Race	White	9						
	Black	8	r = 0.005	r = 0.124	r = 0.063	r = 0.103	r = 0.005	r = 0.078
	Indian	8	p = 0.959	p = 0.198	p = 0.518	p = 0.286	p = 0.960	p = 0.420
	Mixed race	3						
Work experience	≤ 5 years	59	r = 0.027	r = −0.045	r = 0.103	r = 0.029	r = 0.046	r = −0.143
	> 5 years	50	p = 0.778	p = 0.640	p = 0.288	p = 0.766	p = 0.633	p = 0.138

*N* = 109.

## Discussion

Physical activity holds important benefits for physical and mental health and it reduces the risk of metabolic, cardiovascular and many other diseases. The results of the present study revealed low levels of physical activity amongst Estcourt Hospital health professionals when assessed using the GPAQ. There was a strong statistically significant relationship between level of physical activity and age (*p* = 0.000, *r* = −0.637). As the participants’ ages increased, physical activity decreased, which means that older health professionals were physically inactive compared with younger professionals. There was no statistically significant relationship between level of physical activity and other demographics (gender, race and work experience). The number of participants in most race groups (white, Indian and mixed race) was too small to make meaningful conclusions from the correlation results.

Singh and Purohit (2012) reported similar problem of physical inactivity amongst 324 dental healthcare professionals in India. They found that most (68%) dental professionals were minimally involved in physical activity, at < 600 MET-minutes per week. According to Skaal and Pengpid (2011), medical staff and non-medical staff in a tertiary hospital in South Africa showed no significant difference in their lifestyle following an intervention to improve physical activity. Their study showed that the majority of the staff, of whom most were female, fell into the low fitness category, whilst a few demonstrated a moderate level of fitness. A quarter (25%) of participants met the criteria for a high level of fitness, of whom most were male and < 40 years old (Skaal & Pengpid 2011). Suija *et al.* (2010) reported results amongst Estonian family doctors that were contrary to the local relationship between age and physical activity; they found no statistically significant relationship between age and level of physical activity.

Health professionals are considered and expected to be role models to communities for healthy lifestyles, yet they have a poor record of involvement in physical activity. The findings of the current study were not suprising as Jonsdottir *et al.* (2011) also found that healthcare workers follow relatively unfavourable lifestyles and were not involved in healthy lifestyle programmes in their hospitals or their communities. A few studies in other countries have reported results that are contrary to local levels of physical activity amongst health professionals. Medical students in the USA (Frank *et al.* 2008) and physicians of the Minnesota Medical Association (Hensrud *et al.* 1992), to mention a few, presented with higher levels of physical activity than the general population.

Poor physical activity amongst health professionals seems to pose a major threat in their lives, as observed amongst the general population in many studies. Levels of physical activity amongst the young and adults seem to have shown a steady decline in the last few decades in many countries (French, Story & Jeffery 2001; Reilly *et al.* 2004). According to Bozo, Pano and Citozi (2013), office employees in Albania engaged in insufficient physical activity of <600 MET-minutes per week as reported by health professionals in many studies. Amongst the participants, women were found to be more physically inactive than men (Bozo *et al.* 2013).

Studies show that physical inactivity does not only affect certain types of people, but that it is a worldwide problem, including South Africa. According to a study conducted as part of a world health survey in South Africa in 2003, less than one-third of South Africans met the health recommendation of doing at least moderate physical activity for 30 minutes on most days, but preferably every day (Lambert & Kolbe-Alexander 2005). The results further showed that nearly half of all South Africans, and mostly women, were inactive; this implies a considerable general health problem, including health professionals, paradoxically. Oyeyemi *et al.* (2013) reported different results amongst adults in Maiduguri in Nigeria, where 68.6% of adults were found to be adequately physically active. No significant difference was found between men and women; however, physical activity declined as participants’ ages incresed.

In general, people who regularly exercise are healthier than those who do not, with the former benefiting not only physically but also mentally (WHO 2004). As physical inactivity poses a health risk, there is a need to aggressively implement and monitor physical activity promotion programmes for health professionals. The workplace should be used as a setting for interventions that promote physical activity amongst employees because it is where employees spend the most time. Physical activity programmes should be linked with healthcare institutions’ objectives and policies in order to be implemented effectively.

At least 150 minutes of moderate, or 75 minutes of vigorous, aerobic activity per week is recommended, according to WHO (2010) standards. These activities should be performed in bouts of at least 10 minutes. For additional health benefits, 300 minutes of moderate, or 150 minutes of vigorous, aerobic activity per week should be done. Muscle strengthening activities should also be done involving major muscle groups on two or more days a week. Work-related physical activity, walking and jogging, as well as sport, fitness and recreational activities, are forms of exercise that can be beneficial, and they need to be promoted and encouraged to reduce the global health problem.

## Conclusion

Most health professionals at Estcourt Hospital engaged in low levels of physical activity, namely work-related physical activity, walking, cycling, and sporting or fitness or recreational activities. Age appeared to be a determining factor in participation in physical activity. Men were involved in vigorous and sport-related activities even though their level of physical activity did not reach recommended levels. The study findings imply that a health-promoting intervention adjusted for age and gender is urgently required to address the risks posed by physical inactivity amongst all health professionals. Promotion of physical activity in particular by physiotherapists, with their expertise in movement for health, is needed.
